# Preadmission beta-blocker use and 30-day mortality among patients in intensive care: a cohort study

**DOI:** 10.1186/cc10085

**Published:** 2011-03-07

**Authors:** Steffen Christensen, Martin Berg Johansen, Else Tønnesen, Anders Larsson, Lars Pedersen, Stanley Lemeshow, Henrik Toft Sørensen

**Affiliations:** 1 Department of Clinical Epidemiology, Institute of Clinical Medicine, Aarhus University Hospital, Olof Palmes Alle, Aarhus N 8240, Denmark; 2 Department of Anaesthesiology and Intensive Care, Aarhus Hospital, Aarhus University Hospital, Nørrebrogade, Aarhus C 8000, Denmark; 3 Department of Anaesthesiology and Intensive Care Medicine, Uppsala University Hospital, Uppsala S-751 85, Sweden; 4 Division of Biostatistics, College of Public Health, Ohio State University, 10th Avenue, M-116 Starling Loving Hall, Columbus, OH 43210, USA

## Abstract

**Introduction:**

Beta-blockers have cardioprotective, metabolic and immunomodulating effects that may be beneficial to patients in intensive care. We examined the association between preadmission beta-blocker use and 30-day mortality following intensive care.

**Methods:**

We identified 8,087 patients over age 45 admitted to one of three multidisciplinary intensive care units (ICUs) between 1999 and 2005. Data on the use of beta-blockers and medications, diagnosis, comorbidities, surgery, markers of socioeconomic status, laboratory tests upon ICU admission, and complete follow-up for mortality were obtained from medical databases. We computed probability of death within 30 days following ICU admission for beta-blocker users and non-users, and the odds ratio (OR) of death as a measure of relative risk using conditional logistic regression and also did a propensity score-matched analysis.

**Results:**

Inclusion of all 8,087 ICU patients in a logistic regression analysis yielded an adjusted OR of 0.82 (95% confidence interval (CI): 0.71 to 0.94) for beta-blocker users compared with non-users. In the propensity score-matched analysis we matched all 1,556 beta-blocker users (19.2% of the entire cohort) with 1,556 non-users; the 30-day mortality was 25.7% among beta-blocker users and 31.4% among non-users (OR 0.74 (95% CI: 0.63 to 0.87)]. The OR was 0.69 (95% CI: 0.54 to 0.88) for surgical ICU patients and 0.71 (95% CI: 0.51 to 0.98) for medical ICU patients. The OR was 0.99 (95% CI: 0.67 to 1.47) among users of non-selective beta-blockers, and 0.70 (95% CI: 0.58 to 0.83) among users of cardioselective beta-blockers.

**Conclusions:**

Preadmission beta-blocker use is associated with reduced mortality following ICU admission.

## Introduction

Beta-blockers are used widely to treat cardiovascular diseases and have been shown to reduce re-infarction rates and mortality following myocardial infarction [1,2]. In patients with chronic heart failure, beta-blockers improve cardiac function and reduce mortality [3,4]. Results from observational studies and randomized controlled trials suggest that beta-blockers may reduce the risk of perioperative cardiac complications and mortality in high-risk patients undergoing major surgery, although this has recently been challenged [5-12].

During critical illness, the whole body's metabolism shifts towards a hypermetabolic state, primarily in terms of increased resting energy expenditure, rapid muscle loss and hyperglycemia [13-15]. This metabolic shift is mediated mainly through a catecholamine surge and sympathetic activation during the early phase of critical illness [16]. Attenuation of the hypermetabolic state has been associated with reduced mortality [16-18]. For instance, blocking of the beta-adrenergic stimulation of the catecholamine surge has been suggested as the underlying biological mechanism for the reduced mortality observed in beta-blocker users hospitalized with severe trauma and burns [18-21].

Most ICU patients also have varying degrees of the systemic inflammatory response syndrome. Key mediators of the cellular immune system have beta-adrenergic receptors [22,23], and *in vitro *studies have suggested a number of potential beneficial immunomodulating effects of beta-blockers [15,24]. Moreover, the rate of cardiovascular complications is high among intensive care unit (ICU) patients [25,26], and a large proportion have cardiovascular comorbidities.

For these reasons preadmission beta-blocker use may be associated with improved prognosis among ICU patients. At the same time, however, beta-blocker use may have detrimental effects in patients who need beta-stimulation to maintain adequate tissue perfusion.

Virtually no data exist on the association between beta-blocker use and mortality among general ICU patients. We, thus, examined whether preadmission beta-blocker use was associated with mortality within 30 days of ICU admission.

## Materials and methods

### Setting

We conducted this cohort study based on prospectively collected data obtained from population-based medical databases in northern Denmark. The study population consisted of all patients admitted for the first time to an ICU in one of three hospitals within the Aarhus University Hospital network during the study period. The ICUs are highly specialized multidisciplinary tertiary units serving as both primary and referral ICUs, and together they cover all major medical specialties. The nurse-to-patient ratio is 1:1. For study purposes, two different data collection periods were defined based on the initial availability of computerized ICU data records: 1 January 1999 to 31 December 2005 for patients treated in Aarhus and Skejby Hospitals, and 1 January 2001 to 31 December 2005 for patients treated in Aalborg Hospital.

Since 1968 every Danish citizen has received at birth a unique civil registration number from the Danish Civil Registration System. This number permits accurate linkage across all Danish registries.

### ICU patients

A research database at the University of Aarhus contains data on all admissions to the ICUs at Aarhus, Aalborg and Skejby hospitals, including patient civil registration numbers, dates of ICU admission and discharge, use of mechanical ventilation, and use of renal replacement therapy. We did not include patients who were admitted for planned postoperative observation of less than 24 hours. Patients younger than 45 years of age were also not included because beta-blockers are rarely prescribed to persons in this age group in Denmark. Thus 9,515 ICU patients remained for further analysis. Only patients with complete laboratory data were included in the main analysis. The study cohort thus encompassed 8,087 eligible ICU patients with a first ICU admission during the study period (85% of the entire cohort).

### Preadmission use of beta-blockers

We collected data on all prescriptions filled by study patients since 1997 from a prescription database that contains data, transferred electronically from all pharmacies in the region, on customers' civil registration numbers, types and dosages of drugs prescribed, and redemption dates [27]. We defined current beta-blocker use as at least one filled prescription within 125 days before ICU admission. The 125-day period allowed us to capture most current beta-blocker use, because few beta-blocker prescriptions are expected to last more than 125 days in Denmark. In a sensitivity analysis we redefined current use as redemption of at least one prescription within 60 days before ICU admission. Since including prevalent use in the analysis could introduce bias, we also defined subgroups of "new" and "long-term" current beta-blocker users. "New" users had filled their first-ever beta-blocker prescription *within *125 days of ICU admission, and "long-term" users had filled their first-ever prescription *more than *125 days before ICU admission [28]. We also categorized patients according to the type of the last beta-blocker prescribed before ICU admission (that is, non-selective (Anatomical Therapeutic Chemical (ATC)-codes: 'C07AA02' 'C07AA03' 'C07AA05' 'C07AA06' 'C07AA07' 'C07AA16'); non-selective combined with alpha-adrenergic blocker (ATC codes 'C07AG01' 'C07AG02'); cardioselective (ATC codes 'C07AB02' 'C07AB03' 'C07AB04' 'C07AB05' 'C07AB07' 'C07AB09').

### Other prognostic factors

We used the Danish National Patient Registry (DNPR) to identify the primary diagnosis, that is, the diagnosis listed first in the hospital registry record for the admission during which a patient was transferred to the ICU [29]. On the basis of the primary diagnosis, patients were grouped into eight disease categories: infectious diseases; endocrinology (including diabetes); cardiovascular diseases; respiratory diseases; gastrointestinal and liver diseases; cancer; trauma and poisoning; and others. We classified patients as 'medical' or 'surgical' according to whether they had undergone any surgery within seven days prior to ICU admission. To control for comorbidity, we computed a Charlson Comorbidity Index (CCI) score, based on a patient's entire previous hospital history since 1977. We defined three comorbidity levels based on the CCI: low (score of 0), medium (score of 1 to 2), and high (score ≥ 3) [30]. Alcoholism-related disease was defined as either a previous hospital diagnosis of an alcoholism-related disease (for example, alcoholic liver disease) or redemption of a prescription for disulfiram. Through the DNPR we also obtained data on use of temporary pacemakers, isoprenaline, vasopressors, and inotropic drugs in 2004 and 2005. We retrieved information on filled prescriptions for other cardiovascular drugs including angiotensin-converting enzyme (ACE-) inhibitors, statins and low-dose aspirin. From hospital laboratory databases we obtained data on the lowest hemoglobin measurement, the highest white blood cell count (WBC), and the highest levels of C-reactive protein (CRP) and creatinine registered within two days before or after ICU admission. We were able to retrieve all laboratory data for 8,087 patients (85% of the entire cohort). We obtained data on urbanization and marital status at the time of ICU admission from the Danish Civil Registration System (CRS), as a measure of social status [31].

### Mortality and migration data

To assess deaths and migration in our patient cohort, we accessed data from the CRS [31]. The CRS contains information for the entire Danish population on migration and changes in vital status, including exact date of death, updated on a daily basis.

### Statistical analysis

Follow-up began on the date of the first ICU admission and continued until death, migration, or 30 days following ICU admission, whichever came first. We computed life table estimates for mortality within 30 days.

We used logistic regression to compute the odds ratio (OR) of death for users of beta-blockers compared with non-users, controlling for covariates listed in Table 1. To assess the influence of excluding ICU patients with missing laboratory data from the analysis, we repeated the logistic regression model including all 9,515 ICU patients, controlling for all covariates listed in Table 1, except laboratory data.

**Table 1:** Characteristics of ICU patients, Aarhus University Hospital, 1999 to 2005

	Overall		Propensity-score-matched cohorts	
	**No current use of beta-blockers**	**Current use of beta-blockers**	**No current use of beta-blockers**	**Current use of beta-blockers**
	**N (%)**	**N (%)**	**N (%)**	**N (%)**

***Overall***	6,531 (80.8%)	1,556 (19.2%)	1,556 (0%)	1,556 (100%)
***Age group***				
**46 to 59**	2,100 (32.2%)	344 (22.1%)	307 (19.7%)	344 (22.1%)
**60 to 75**	2,880 (44.1%)	740 (47.6%)	790 (50.7%)	740 (47.6%)
**75+**	1,551 (23.7%)	472 (30.3%)	459 (29.5%)	472 (30.3%)
***Gender***				
**Female**	2,796 (42.8%)	598 (38.4%)	623 (40.0%)	598 (38.4%)
**Male**	3,735 (57.1%)	958 (61.6%)	933 (60.0%)	958 (61.6%)
***Diagnostic category***				
**Infectious disease**	152 (2.3%)	37 (2.4%)	22 (1.4%)	37 (2.4%)
**Cancer**	1,200 (18.4%)	143 (9.2%)	114 (7.3%)	143 (9.2%)
**Diabetes**	88 (1.4%)	18 (1.2%)	14 (0.8%)	18 (1.2%)
**Cardiovascular**	1,812 (27.7%)	722 (46.4%)	756 (48.6%)	722 (46.4%)
**Respiratory**	753 (11.5%)	117 (7.5%)	103 (6.6%)	117 (7.5%)
**Gastrointestinal**	797 (12.2%)	176 (11.3%)	196 (12.6%)	176 (11.3%)
**Trauma/poisoning**	834 (12.8%)	137 (8.8%)	160 (10.3%)	137 (8.8%)
**Other**	895 (13.7%)	206 (13.2%)	191 (12.3%)	206 (13.2%)
***Surgery within 7 days***				
**No surgery**	2,829 (43.3%)	611 (39.3%)	592 (38.1%)	611 (39.7%)
**Surgery**	3,702 (56.7%)	945 (60.7%)	964 (62.0%)	945 (60.7%)
***Comorbidity***				
***Charlson score***				
**0**	1,880 (28.8%)	237 (15.2%)	224 (14.4%)	237 (15.2%)
**1 to 2**	2,740 (42.0%)	649 (41.7%)	666 (42.8%)	649 (41.7%)
**3 +**	1,911 (29.3%)	670 (43.1%)	666 (42.8%)	670 (43.1%)
***Alcoholism-related disorders***	696 (10.7%)	160 (10.3%)	150 (9.6%)	160 (10.3%)
***Preadmission drug use ACE-inhibitors***	954 (14.6%)	633 (40.7%)	644 (41.4%)	633 (40.7%)
***Statins***	415 (6.4%)	469 (30.1%)	436 (28.0%)	469 (30.1%)
***Low-dose aspirin***	477 (7.3%)	336 (21.6%)	311 (20.0%)	366 (21.6%)
***Marital status***				
**Married**	2,729 (41.8%)	671 (43.1%)	652 (41.9%)	671 (43.1%)
**Never married**	476 (7.3%)	91 (5.7%)	88 (5.7%)	91 (5.9%)
**Divorced**	665 (10.2%)	128 (8.2%)	129 (8.3%)	128 (8.2%)
**Widowed**	910 (13.4%)	246 (15.8%)	243 (15.6%)	246 (15.8%)
**Unknown**	1,751 (26.8%)	420 (27.0%)	444 (28.5%)	420 (27.0%)
Laboratory data*				
***Hemoglobin***				
**Low**	3,049 (47.7%)	805 (51.8%)	771 (49.6%)	805 (51.7%)
**High**	3482 (53.4%)	751 (48.2%)	785 (50.4%)	751 (48.3%)
***Leukocytes***				
**Low**	3,244 (49.7%)	787 (50.6%)	737 (47.4%)	787 (50.6%)
**High**	3,287 (50.3%)	769 (49.4%)	819 (52.6%)	769 (49.4%)
***C-reactive protein***				
**Low**	3,318 (50.8%)	723 (46.8%)	703 (45.2%)	723 (46.8%)
**High**	3,213 (49.2%)	833 (53.2%)	453 (54.8%)	833 (53.2%)
***Creatinine***				
**Low**	3,458 (53.0%)	577 (37.1%)	645 (41.5%)	577 (37.1%)
**High**	3,073 (47.1%)	979 (62.9%)	911 (58.6%)	979 (62.9%)

*Laboratory data were categorized into two groups based on median values.

For the propensity score analysis we generated a multivariable logistic regression model that predicted beta-blocker use among ICU patients based on the covariate profile listed in Table 1 and computed the propensity score (that is, the probability of beta-blocker use) for all ICU patients. Using a greedy matching algorithm, we matched each beta-blocker user with the one non-user with the closest propensity score, within a maximum matching range of ± 0.025. In this manner we were able to match each beta-blocker user to a non-user. Propensity score matching decreased the absolute standardized differences of each covariate to values below 0.1, indicating that an adequate balance was achieved. We used conditional logistic regression to compute the odds ratio (OR) as a measure of relative risk of death within 30 days after ICU admission for beta-blocker users compared with non-users in the propensity score-matched cohort. We conducted separate analyses for subgroups defined according to admitting department, use of mechanical ventilation, type of surgery, diagnostic category, renal replacement therapy, use of selective vs. non-selective beta-blockers, and new vs. long-term beta-blocker use. We also repeated the propensity score-matched analysis using conditional logistic regression analysis to control for all covariates included in the model. To assess possible unmeasured confounding by indication for beta-blockers (primarily cardiovascular diseases treated by general practitioners and thus not registered in the DNPR), we restricted an analysis to patients previously hospitalized with cardiovascular diseases or diabetes. Finally, we repeated the propensity-score-matched analysis after defining current beta-blocker use as redemption of at least one prescription within 60 days before ICU admission.

We calculated the biological interaction - or effect measure modification - between statin and beta-blocker use as the mortality in patients who used both statins and beta-blockers, minus the mortality that statin use adds, minus the mortality that beta-blocker use adds, and minus the mortality in patients using neither beta-blockers nor statins. We also conducted a propensity score-matched analysis stratified by statin use.

All analyses were performed using SAS version 9.1.3 (SAS Institute Inc., Cary, NC, USA).

The study was approved by the Danish Data Protection Agency and the Aarhus University Hospital Registry Board. Data were obtained from Danish registries, which are generally available to researchers and their use does not require informed consent.

## Results

### Descriptive data

Out of 8,087 ICU patients with complete laboratory data available, 1,556 (19.2%) were current users of beta-blockers upon ICU admission (Table 1). The most commonly used beta-blocker was metoprolol (*n *= 986 (63.4%)). Beta-blocker users were older than non-users and had higher levels of comorbidity (43.1% of beta-blocker users had a CCI score > 3 vs. 29.3% of non-users). Compared with non-users, beta-blocker users were admitted more often with cardiovascular diseases and less often with cancer or following trauma/poisoning. As expected, beta-blocker users compared to non-users were more often users of other cardiovascular drugs including statins (30.1% vs. 6.4%), ACE-inhibitors (40.7% vs. 14.6%), and low-dose aspirin (21.6% vs. 7.3%). Propensity score matching balanced out these differences between the two groups (Table 1).

Among ICU patients admitted in 2004 and 2005, 24 (1.2%) beta-blocker users and 10 (1.8%) non-users were treated with a temporary pacemaker. Isoprenaline was used for nine (0.4%) among beta-blocker uses and four (0.7%) among non-users. Vasopressors and inotropic drugs were used in 703 (36.5%) and 189 (9.3%) of beta-blocker users vs. 211 (38.1%) and 59 (10.7%) of non-users.

### Mortality

**Figure 1:**
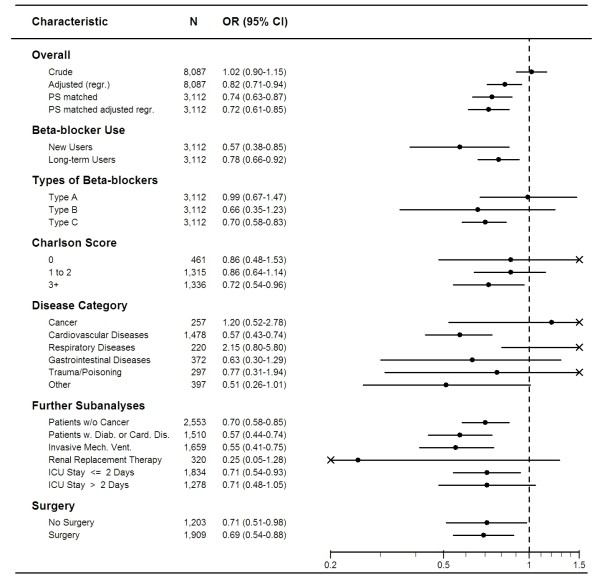
**Odds ratios (ORs) for death within 30 days after ICU admission among beta-blocker users vs. non-users**. Type A: Non-selective beta-blockers, Type B: non-selective beta-blockers combined with alpha-adrenergic blockers, type C: cardioselective beta-blockers).

Inclusion of all 8,087 ICU patients in a logistic regression analysis yielded an adjusted OR of 0.82 (95% CI: 0.71 to 0.94) for beta-blocker users compared with non-users. In the propensity score-matched analysis of 1,556 beta-blocker users and 1,556 non-users, mortality was 25.7% among beta-blocker users and 31.4% among non-users (corresponding to an OR of 0.74 (95% CI: 0.63 to 0.87)) (Figure 1).

Among users of non-selective beta-blockers, the estimated OR was 0.99 (95% CI: 0.67 to 1.47); among users of non-selective beta-blockers combined with alpha-adrenergic blockers, the estimated OR was 0.66 (95% CI: 0.35 to .23); for users of cardioselective beta-blockers the estimated OR was 0.70 (95% CI: 0.58 to 0.83). We observed decreased ORs in most diagnostic categories, except for patients admitted with cancer or respiratory diseases; however, the relatively small number of patients in these categories resulted in risk estimates with low statistical precision. Among patients treated with invasive mechanical ventilation, the OR was 0.55 (95% CI: 0.41 to 0.75), and among those treated with renal replacement therapy, the OR was 0.25 (95% CI: 0.05 to 1.28).Using a 60-day exposure window, rather than a 125-day window, to define current beta-blocker use yielded an OR of death of 0.74 (95% CI: 0.60 to 0.91). For new users, the OR of beta-blocker use was 0.57 (95% CI: 0.38 to 0.85) and for long-term users, the OR of beta-blocker use was 0.78 (95% CI: 0.66 to 0.92). When the analysis was restricted to patients with diabetes or cardiovascular comorbidities, the OR was 0.57 (95% CI: 0.44 to 0.74).

The biological interaction between beta-blocker and statin uses was -11.7% (the mortality in beta-blocker users who also used statins (21.7%), minus the estimated 25.4% mortality among those who used neither beta-blockers nor statins, minus the estimated 0.6% (26.0%-25.4%) mortality among statin-only users, minus the estimated -5.8% (21.7%-27.5%) mortality among beta-blocker users who died due to beta-blocker use). The negative value suggests an additional beneficial effect - that is, a mortality reducing effect - from combining beta-blockers and statins. Like the analysis of absolute mortality described above, the propensity score matched analysis stratified by statin use also suggested that combining statins and beta-blockers had an even further beneficial mortality-reducing effect compared with beta-blocker monotherapy (OR = 0.51 (95% CI: 0.35 to 0.73) among beta-blocker users who also used statins vs. OR = 0.89 (95% CI: 0.73 to 1.08)) among beta-blocker users who did not use statins).

## Discussion

In this cohort study we found that preadmission use of beta-blockers was associated with reduced mortality during the 30 days following ICU admission. To our knowledge this is the first study of the association between beta-blocker usage and mortality in general ICU patients, although a number of studies have examined beta-blocker use in patients with specific diseases or undergoing major surgery routinely requiring postoperative ICU admission. Several studies reported that beta-blocker use may reduce perioperative mortality in patients undergoing major non-cardiac surgery, but no earlier study provided separate data for ICU patients [5,8,9,32].

In contrast with our findings, the POISE randomized controlled trial reported that acute administration of high-dose beta-blocker therapy perioperatively was associated with reduced risk of myocardial infarction, but increased risk of total mortality [10]. Of note, sepsis and other infections were more common causes of death among beta-blocker users than among non-users, while there was no difference in risk of death due to multiple organ failure, cardiogenic shock, or heart failure between users and non-users. Less than 30% of POISE participants were transferred to an ICU. In line with our findings, a US observational study of 4,117 trauma patients found that beta-blocker use was associated with reduced in-hospital mortality [19]. The authors speculated whether beta-blocker use led to attenuation of the detrimental effects of hypermetabolism and increased tissue oxygen consumption related to severe trauma. Beta-blocker use has also been reported to have beneficial effects in patients with severe burns, apparently by decreasing energy expenditure and muscle catabolism [18,20].

Our large population of general ICU patients permitted robust estimates in a substantial number of predefined patient categories. Use of prospectively recorded data from independent medical databases with complete follow-up limited the risk of selection, information and surveillance biases. The completeness and nature of the prescription database ensured that measurement of preadmission beta-blocker use was virtually complete [27]. Of note, changing the length of the period defining exposure from 125 days to 60 days before ICU admission had virtually no impact on our risk estimates. Thus, the influence of bias arising from misclassification of beta-blocker use due to the length of the period defining exposure as well as from unmeasured time bias should be minor [33,34].

A potential study weakness is the lack of random assignment of beta-blocker use, which may have introduced confounding. Beta-blockers are prescribed for cardiovascular diseases that may be associated with increased mortality in ICU patients. Confounding arising from underlying cardiovascular diseases, therefore, would be most likely to attenuate our relative risk estimates towards the null. Some patients with an indication for beta-blocker treatment may not have been treated, for several reasons: because their condition was undiagnosed, because treatment was stopped due to side effects, or because of poor compliance. This potentially could have influenced our results. We controlled for a wide range of covariates using both propensity score-matched analysis and logistic regression analysis. Beta-blocker use was associated with reduced mortality in analyses restricted both to patients with cardiovascular comorbidities and to patients admitted with cardiovascular diseases as the primary reason for hospitalization. Thus, uncontrolled confounding by indications for beta-blocker use is unlikely to explain our findings. Still, any lack of specificity in routinely recorded data may have reduced our ability to completely remove confounding. We lacked data on severity of illness, such as SAPS, SOFA, or APACHE scores; however, laboratory data as well as use of vasopressors and inotropics was virtually the same among beta-blocker users and non-users. We found consistent mortality reducing effects from beta-blocker use in analysis restricted to ICU patients treated with mechanical ventilation and renal replacement therapy further indicating that confounding by severity of disease seems unlikely to explain our findings.

Based on our study's observational data we may only speculate on the extent to which the immune-modulating effects, cardioprotective effects, and attenuation of the hypermetabolic state of critical illness may explain our findings. In asthma patients long-term beta-blocker use has been reported to result in upregulation of beta-receptors [35]. This may be beneficial in ICU patients who require beta-stimulation to maintain adequate tissue perfusion. Our results may, therefore, not hold for patients who start taking beta-blockers after getting severely ill. Of note, however, we found slightly more pronounced mortality reductions in new beta-blocker users compared with long-term users. Still, since we had no data on in-hospital beta-blocker use, we could not address the question of whether beta-blocker use initiated immediately before ICU admission is associated with mortality. The mortality reducing effect of combining statins and beta-blockers, that is, the biological interaction, could not solely be explained by addition of the mortality reducing effect of beta-blockers and statins. However, we know of no biological mechanism explaining an additive beneficial effect from combining statins and beta-blockers. Also, since the study was not primarily designed to study the interaction between statins and beta-blockers potential confounding factors may not have been included in the analysis and uncontrolled confounding may thus have influenced the results. We do, however, believe the risk to be minor. Still, it remains to be fully elucidated whether an additional beneficial effect of combining beta-blockers and statins, compared to beta-blocker monotherapy, is a true biological effect. Our data suggest that the beneficial effect of beta-blocker use may be restricted to cardio-selective beta-blockers. However, the relatively low number of non-selective beta-blocker users produced statistically imprecise mortality estimates, hindering any clear interpretation of the results in these subgroups.

A concern is that some potentially beneficial effects of beta-blockers in critically ill patients may be outweighed by a decreased oxygen supply and decreased tissue perfusion associated with reduced cardiac output [21]. Another concern is the well-known side effects of beta-blockade including severe hypotension and bradycardia; however, in the present study the use of a temporary pacemaker, isoprenaline as well as vasopressors and inotropic drugs were virtually the same among beta-blocker users and non-users. Although these data provide only a rough measure of severe side effects of beta-blocker use, they support the overall safety of this medication.

## Conclusions

In conclusion, preadmission beta-blocker use is associated with reduced 30-day mortality in general ICU patients.

## Key messages

• Beta-blocker use is associated with reduced risk of death within 30 days in general ICU patients.

• The mortality reducing effect was similar among medical and surgical ICU patients and was not restricted to patients admitted with cardiovascular diseases.

• It remains to be fully elucidated whether an additional beneficial effect of combining beta-blockers and statins, compared to beta-blocker monotherapy, is a true pharmacological effect.

## Abbreviations

ACE: angiotensin-converting enzyme; ATC: anatomical therapeutic chemical; CCI: Charlson Comorbidity Index; CRP: C-reactive protein; CRS: civil registration system; DNPR: Danish National Patient Registry; OR: odds ratio; WBC: white blood cell count.

## Competing interests

The authors declare that they have no competing interests.

## Authors' contributions

SC, AL, ET and HTS conceived the study idea. SC, MBJ, SL and HTS designed the study. LAP, HTS and MBJ collected the data. MBJ, SL and LP analysed the data. All authors interpreted the findings. SC, ET and AL reviewed the literature. SC wrote the first draft and all authors edited the manuscript and approved the final version.
